# High Tyrosol and Hydroxytyrosol Intake Reduces Arterial Inflammation and Atherosclerotic Lesion Microcalcification in Healthy Older Populations

**DOI:** 10.3390/antiox13010130

**Published:** 2024-01-22

**Authors:** Nada Zoubdane, Redha-Alla Abdo, Michel Nguyen, M’hamed Bentourkia, Eric E. Turcotte, Hicham Berrougui, Tamas Fulop, Abdelouahed Khalil

**Affiliations:** 1 Geriatrics Unit, Department of Medicine, Faculty of Medicine and Health Sciences, University of Sherbrooke, Sherbrooke, QC J1H 4N4, Canada; nada.zoubdane@usherbrooke.ca (N.Z.); redha-alla.abdo@usherbrooke.ca (R.-A.A.); hicham.berrougui@usherbrooke.ca (H.B.); tamas.fulop@usherbrooke.ca (T.F.); 2 Cardiology Unit, Department of Medicine, Faculty of Medicine and Health Sciences, University of Sherbrooke, Sherbrooke, QC J1H 4N4, Canada; michel.nguyen@usherbrooke.ca; 3 Department of Nuclear Medicine and Radiobiology, University of Sherbrooke, Sherbrooke, QC J1K 2R1, Canada; mhamed.bentourkia@usherbrooke.ca; 4 Sherbrooke Molecular Imaging Center (CIMS), 3001, 12th Ave N., Sherbrooke, QC J1H 5NY, Canada; eric.e.turcotte@usherbrooke.ca

**Keywords:** atherosclerosis, positron emission tomography, ^18^F-FDG/^18^F-NaF, vascular inflammation, aging, tyrosol, hydroxytyrosol

## Abstract

Aging is an important risk factor for cardiovascular diseases and convincing data have shown that chronic low-grade inflammation, which develops with advanced age, contributes significantly to cardiovascular risk. The present study aimed to use ^18^F-FDG/^18^F-NaF-PET/CT imaging to, respectively, gauge arterial inflammation and microcalcification in a healthy elderly population and to assess the potential benefits of a tyrosol- and hydroxytyrosol-rich diet on these two markers of atherosclerotic plaque fragility. Eleven healthy participants (mean age 75 ± 5.67 years) were supplemented for 6 months with high polyphenol-rich extra virgin olive oil (HP-EVOO), extra virgin olive oil (EVOO), or refined olive oil (ROO). The participants underwent PET/CT imaging with ^18^F-FDG and ^18^F-NaF radiotracers at baseline and after 6 months. ^18^F-FDG and ^18^F-NaF uptakes were quantified using standardized uptake values (SUV) and were categorized based on artery calcification and olive oil type. A total of 324 slices of the aortas of the imaged participants were analyzed for arterial inflammation and 327 slices were analyzed for microcalcification. ^18^F-FDG uptake was significantly higher in the non-calcified segments than in the calcified segments (SUVmax = 2.70 ± 0.62 and SUVmax = 2.54 ± 0.44, respectively, *p* < 0.042). Conversely, the non-calcified segments displayed significantly lower ^18^F-NaF uptake than the calcified segments (SUVmax = 1.90 ± 0.37 and 2.09 ± 0.24, respectively, *p* < 0.0001). The 6-month supplementation with HP-EVOO induced a significant reduction in ^18^F-FDG uptake in both the non-calcified (2.93 ± 0.23 to 2.75 ± 0.38, *p* < 0.004) and calcified segments of the aortas (2.25 ± 0.29 to 2.15 ± 0.19, *p* < 0.02). ^18^F-NaF uptake was also significantly lower in patients supplemented with HP-EVOO (SUVmax = 1.98 ± 0.33 at baseline compared to 1.85 ± 0.28, after the 6-month supplementation, *p* < 0.004), whereas no significant effect was observed with EVOO. Conversely, participants supplemented with ROO displayed a significant increase in ^18^F-NaF uptake (SUVmax = 1.78 ± 0.34 to 1.95 ± 0.34, *p* < 0.0001). The present study confirmed that a phenolic-compound-rich diet reduces both arterial inflammation and atherosclerotic lesion microcalcification and demonstrated that ^18^F-FDG/^18^F-NaF-PET/CT imaging is a valuable approach for assessing age-related arterial damage.

## 1. Introduction

Cardiovascular disease (CVD) is one of the leading causes of death globally and is the major cause of mortality and morbidity in seniors [1]. Atherosclerotic CVD is caused by the disruption of atherosclerotic plaque and thrombus formation, which can cause a stroke or myocardial infarction [2]. Atherosclerosis is a chronic inflammatory disease in which the arteries become clogged with fatty plaque. Inflammation is present at all stages of the atherosclerosis process, from the initiation of atherosclerotic plaque formation to plaque disruption and thrombosis [3]. Inflammaging, a chronic low-grade inflammation that occurs with aging, is implicated in the development and progression of atherosclerosis [3,4]. Thomas et al. proposed a set of characteristics that define “ideal” biomarkers that encompass a range of molecules that indicate various disease states and that can be used to measure inflammation [5]. Although biomarkers are convenient and cost-effective for measuring systemic inflammation, they lack specificity when it comes to assessing arterial inflammation. Arterial inflammation is localized inflammation that occurs in the arteries and that significantly contributes to a detrimental cycle that leads to microcalcification and, ultimately, results in plaque rupture [6]. Despite this, arterial inflammation is often overlooked and has not been extensively studied.

Arterial calcification is a marker of sub-clinical atherosclerosis and a predictor of future cardiovascular events, particularly microcalcification, which correlates with plaque fragility. Although oxidative stress is considered to be an important factor in the formation of atherosclerosis-associated calcification, vitamin antioxidant intake does not appear to reduce arterial calcification [7]. Conversely, omega-3 polyunsaturated fatty acid supplementation was associated with a reduction in plaque calcification [8]. Polyphenols and particularly epigallocatechin gallate and resveratrol also reduced arterial calcification in a mouse model [9,10].

Positron emission tomography (PET) combined with computed tomography (CT) is a highly sensitive non-invasive technique that is now used to localize and quantify arterial inflammation and to assess atherosclerotic plaque stability [11]. The arterial uptake of ^18^F-fluorodeoxyglucose (^18^F-FDG), which is the most commonly used radiotracer, is associated with an increase in the macrophage burden and inflammatory gene expression [12,13]. Moreover, arterial inflammation is ultimately associated with arterial microcalcification. Proinflammatory cytokines induce, among other changes, the osteogenic transformation of vascular smooth muscle cells that gives rise to microcalcification, which is associated with an increase in atherosclerotic plaque fragility [14]. ^18^F-sodium-fluoride (^18^F-NaF) has emerged as a new radiotracer for assessing vascular microcalcification (<50 μM) which is below the limit of the resolution of CT. Previous studies, including ours, have shown that PET/CT using ^18^F-FDG and/or ^18^F-NaF can localize arterial inflammation [15], to detect the early stages of atherosclerotic plaque development [16] and to investigate the reduction of arterial inflammation by some drugs [17].

Healthy dietary habits have been shown to improve cardiovascular health and reduce the risk of cardiovascular events. The olive-oil-centered Mediterranean diet has also been associated with a lower incidence of major CVD events [18]. Phenolic compounds in olive oil, particularly tyrosol (Tyr) and hydroxytyrosol (HTyr), have emerged as key components responsible for the health benefits of the Mediterranean diet. We have previously shown that these phenolic compounds help protect against the development of CVD by regulating HDL cholesterol efflux capacity, reducing LDL atherogenicity [19], and modulating monocyte cytokine secretion in high-risk CVD patients [20]. Several other studies have shown that Tyr and HTyr alleviate oxidative stress [21] and protect against LDL peroxidation, which is the first step in the atherosclerosis process [22,23]. Tyr and HTyr can also effectively attenuate the release of pro-inflammatory cytokines, chemokines, and growth factors, including IL-6, IL-8, CXCL13, and vascular endothelial growth factor, and suppress molecules capable of recruiting inflammatory monocytes and activating macrophages, potentially mitigating the inflammatory response [24]. This suggests that Tyr and HTyr may exert anti-atherosclerotic effects by modulating inflammation via the inhibition of key inflammatory signaling pathways [25]. However, to our knowledge, no studies have investigated the effect of Tyr and HTyr in healthy seniors who, due to the occurrence of inflammaging, may be at high risk for developing atherosclerosis and CVD [26]. The present study thus aimed to gauge the involvement of arterial inflammation in microcalcification in healthy seniors and to assess the effect of a Tyr- and HTyr-rich diet on these two determinants of CVD risk.

## 2. Materials and Methods

### 2.1. Participant Recruitment

The present study was conducted within the framework of the ongoing LIPIMAGE cohort, a prospective study employing PET imaging to investigate the impact of EVOO on the progression and stability of atherosclerotic plaque in high-risk CVD patients. The research protocol adhered to the guiding principles of the Helsinki Declaration and received approval from the Ethics Committee of the University of Sherbrooke (Approval Number: 2019-3145). All participants provided written informed consent before being admitted to the study.

Eleven healthy individuals (5 women and 4 men, mean age: 75 ± 5.67 years) were selected for the present study based on specific inclusion/exclusion criteria. Inclusion criteria of the participants included: over 65 years of age, healthy, no clinical signs of CVD, no recent or familial medical history of note, and having electrocardiograms (ECG). Exclusion criteria included: diabetes, chronic inflammatory disorders, chronic kidney illness (serum creatinine level > 250 μmol/L), cancer, and taking immunostimulants or anti-inflammatory drugs. Smokers and participants who regularly consumed EVOO (>3 times a week in the raw form) were also excluded. Participants were asked to maintain the same eating habits through the supplementation period.

### 2.2. Study Protocol

The study protocol comprised two phases conducted over a 6-month period. Each participant underwent a blood test and an initial ^18^F-FDG/^18^F-NaF-PET-CT examination at the Sherbrooke Molecular Imaging Center. The participants were divided into three groups, each of which was instructed to consume 25 mL/day of one of three types of olive oil (high polyphenol extra virgin olive oil (HP-EVOO, 3 participants), extra virgin olive oil (EVOO, 3 participants), or refined olive oil (ROO, 5 participants) for 6 months.

Compliance with the supplementation was monitored at monthly appointments during which the participants completed questionnaires and the total olive oil consumed was assessed by determining the amount of olive oil remaining in the containers. The participants received no specific dietary advice or physical activity recommendations before the study and continued their normal daily activities for the duration of the study. Following the 6-month supplementation period, a second blood test and a second ^18^F-FDG/^18^F-NaF-PET-CT examination were conducted for a comparative analysis.

The blood tests encompassed standard biochemical assessments (glycemia, lipid profiles) and serum markers of inflammation and cardiovascular risk, including CRP, Hs-CRP, SAA, platelet markers, sedimentation rate, and fibrinogen. Plasma cytokine levels (iLβ-1β, IL-6, IL-10, and TNF-α) were assessed using a Luminex with a Human High Sensitivity Kit (#HSTCMAG-28SK-12, Millipore Sigma, Burlington, MA, USA).

### 2.3. Imaging Procedures

Patients were imaged with a Siemens PET/CT nuclear medicine scanner (Siemens Biograph Vision 600 Edge) using ^18^F-FDG and ^18^F-NaF positron-emitting radiotracers as previously described [27] for, respectively, metabolic activity and microcalcification levels. Briefly, a non-enhanced low-dose CT scan to detect arterial calcium accumulation was performed and was followed immediately with a 30 min scan with the ^18^F-NaF and then another 30 min scan with ^18^F-FDG. The scans were taken in dynamic mode and yielded 35 sequential time frames. ^18^F-NaF and ^18^F-FDG were injected intravenously and consisted of a bolus of 112 ± 30 MBq and 160 ± 27 MBq, respectively, depending on the participant’s weight. The two injections were given at a 90 min interval to allow the activity of ^18^F-NaF to decrease before the ^18^F-FDG was injected. A 30 s blank scan was performed prior to injecting the ^18^F-FDG to eliminate any remaining ^18^F-NaF activity.

### 2.4. Image Analyses

For the image analyses, the ^18^F-NaF and ^18^F-FDG were co-registered using the respective CT images. The imaging scan was timed based on the time–activity curve plateau, which was the average of the preceding three-time frames, i.e., 15 min, beginning after 15 min for the ^18^F-NaF injection and 15.5 min for the ^18^F-FDG injection. A calcification threshold of 130 HU in the CT scans was used to identify artery images [28]. The ROI (region of interest) was established on the transaxial slice of the complete artery image using an active contour or manual delineation to define the aorta in the CT and PET images. The PET artery images were corrected for the partial volume effect (PVE) using recovery coefficients obtained from CT and PET images of a cylindrical phantom with seven cylinders (syringes) with different diameters ranging from 4 to 29 mm for the Biograph scanner using a previously described procedure [29]. The standard uptake value (SUV) was calculated from images obtained more than 15 min after the injection to examine the uptake of ^18^F-NaF and the metabolism of ^18^F-FDG. The lean body mass (LBM) factor was used instead of the body mass of the SUV calculation.

### 2.5. Olive Oil Supplementation and Olive Oil Polyphenol Measurements

Three types of olive oil with different polyphenol contents were used. HP-EVOO (*Olivie Plus 30X*) and EVOO were purchased from OLIVIE PHARMA (Atlas Olive Oil, Casablanca, Morocco). HP-EVOO has a very high polyphenol content, especially of Tyr and HTyr, compared to regular EVOO. The enrichment of polyphenols is induced naturally by growing a specific type of olive tree in very arid areas (rocky desert, with a summer temperature of 52 °C). ROO is an olive oil that is marketed in food stores across Quebec, Canada. It was chosen because it is virtually polyphenol-free.

Tyr and HTyr were analyzed and certified by the Biotechnology Laboratory of the Faculty of Sciences, Dhar El Mehraz (Fez, Morocco). The total phenolic content of each oil was determined using the Folin–Ciocalteu reagent (OD 750 nm, Hitachi UH5300 spectrophotometer).

### 2.6. Statistical Analysis

Data are expressed as means ± standard deviation (SD). A Student’s t-test and nonparametric tests were used when appropriate to determine statistically significant differences. An ANOVA (analysis of variance), followed by a Tukey’s multiple comparisons test, was used to compare more than two groups at baseline and after the 6-month supplementation period. *p* < 0.05 was considered statistically significant. The statistical analyses were performed using GraphPad Prism version 9.5.1 (GraphPad Software, Inc., San Diego, CA, USA).

## 3. Results

The biochemical and anthropometric data of each of the participants in the three groups are presented in Table 1. The groups were comparable with respect to the different biochemical and clinical markers measured (lipid profile, liver function, cardiac function). The CRP level was close to 3 mg/L for some patients, reflecting a low-grade inflammation associated with age. Importantly, throughout the 6-month supplementation period, no matter which olive oil was used, there were no discernible changes in the overall clinical and biochemical profiles compared to baseline or between groups (Table 1).

**Figure 1:**
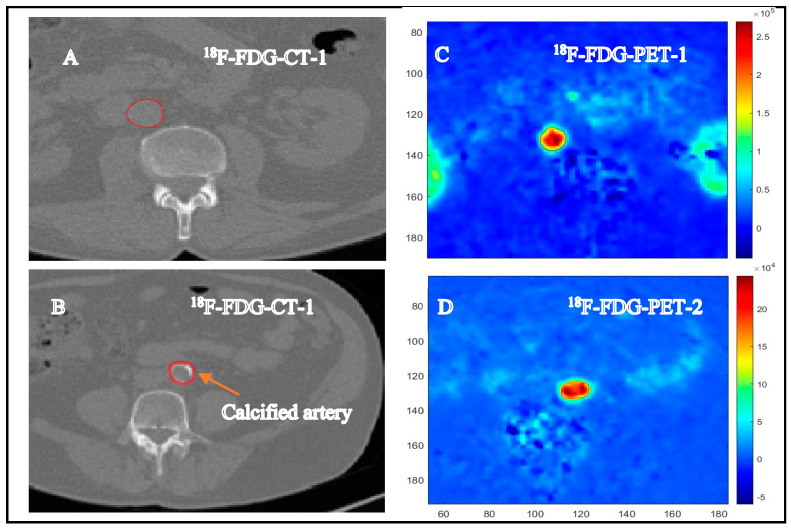
Transaxial PET/CT images of the aortas from one patient. Panels (**A**,**C**) portray the same arterial slice, which is devoid of CT-detected calcification. The PET scan in panel C detected significant ^18^F-FDG uptake. The corresponding CT and PET-^18^F-FDG of a calcified artery are, respectively, shown in (**B**,**D**). The arrow designates the calcified segment within the aorta.

Figure 1 presents transaxial PET/CT images of a participant displaying non-calcified (Figure 1A) and calcified segments of the artery (Figure 1B). Despite their good health status and the absence of any clinical signs of cardiovascular disease, the participants in each of the three groups had aorta segments with no apparent calcification (coronary artery calcification, CAC = 0) as well as aorta segments displaying different levels of calcification (CAC = 1 to 4). Figure 1C,D are the 18F-FDG PET images corresponding to Figure 1A,B, respectively.

**Figure 2:**
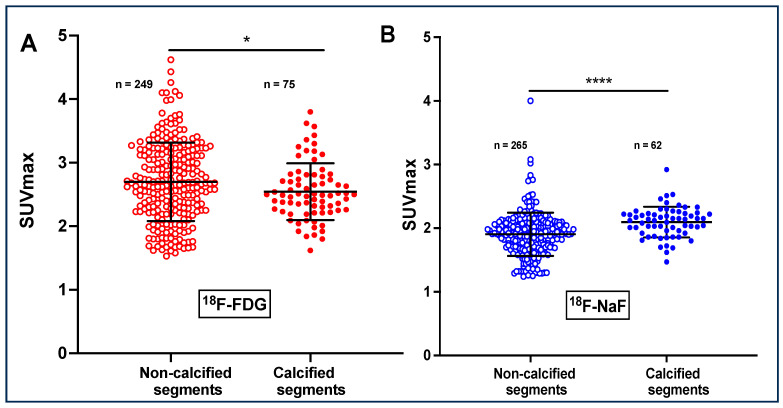
Comparison of the SUVmax values of calcified and non-calcified slices. Panel (**A**) SUVmax for ^18^F-FDG and panel (**B**) for ^18^F-NaF in calcified compared to non-calcified slices of the aorta. Values were determined at baseline for each participant. The data were obtained from transaxial image slices. The letter n is the number of artery slices evaluated for ^18^F-FDG or ^18^F-FDG uptake. **** *p* < 0.0001 and * *p* < 0.04.

The co-registration of the CT and PET images showed that ^18^F-FDG uptake is conspicuous in both non-calcified and calcified arteries, as can be seen in Figure 2. To quantify ^18^F-FDG uptake levels in non-calcified and calcified arteries, a total of 324 slices of the aorta of the 11 imaged participants were analyzed. Of these slices, 249 (76.85%) were apparently healthy without calcification and 75 (23.14%) were calcified. The non-calcified slices showed a significantly higher ^18^F-FDG uptake (SUVmax = 2.70 ± 0.62) than that of the calcified slices (2.54 ± 0.44, *p* <0.04) (Figure 2A). Two patients were excluded because of the higher degree of calcification in their aortas or because data on the radioactivity counts during the PET imaging were missing.

The analyses of the CT and PET images showed that the ^18^F-NaF radiotracer is trapped in the arterial wall both in non-calcified and calcified slices of the aorta. Of the 327 slices analyzed, 265 (81.04%) showed no visible calcification on CT images, whereas 62 slices (18.96%) presented significant calcification. The non-calcified slices presented a significantly lower ^18^F-NaF uptake compared to the calcified slices (SUVmax = 1.90 ± 0.37 and 2.09 ± 0.24, respectively, *p* < 0.0001) (Figure 2B).

Table 2 presents the levels of phenolic compounds in each of the three olive oils used for the supplementation. ROO (control oil) was almost polyphenol-free, whereas HP-EVOO was five times richer in total phenolic compounds than EVOO. Total Tyr and HTyr levels were also significantly higher in HP-EVOO than in EVOO (20 and 30 times higher, respectively).

**Figure 3:**
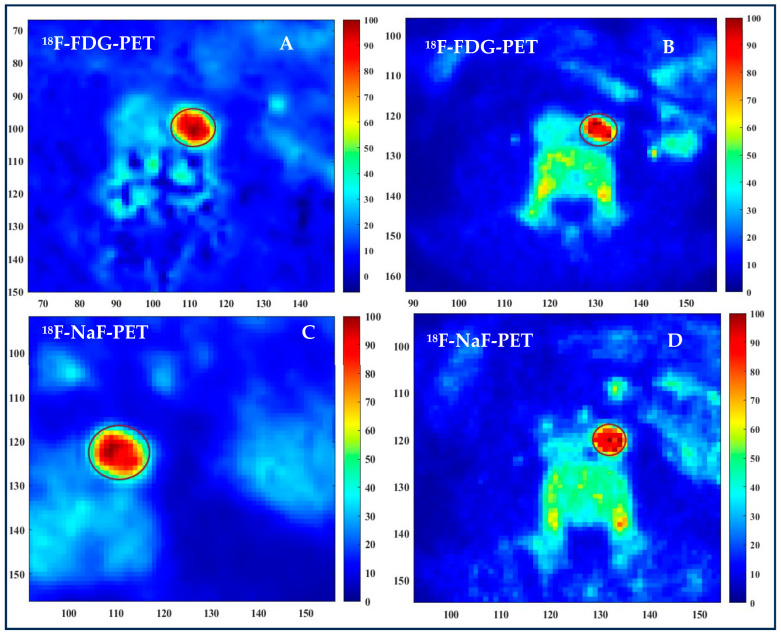
Effects of a 6-month supplementation with HP-EVOO intake on arterial inflammation and atherosclerotic lesion microcalcification. Panel (**B**), compared to panel (**A**), shows a decrease in ^18^F-FDG uptake after 6-month supplementation with HP-EVOO. Panel (**D**), compared to panel (**C**), shows a decrease in ^18^F-NaF uptake after 6-month supplementation with HP-EVOO.

Figure 3 shows ^18^F-FDG and ^18^F-NaF uptake in the aorta at baseline and after the 6-month supplementation with HP-EVOO. The color scale represents the percentage of SUVmax levels, and a noticeable decrease in visual intensity is observed, suggesting a reduction in 18F-FDG and 18F-NaF uptake in the aorta following the 6-month HP-EVOO supplementation. The reduction in the number of pixels with 100% SUVmax is clearly visible when comparing panel A to B and panel C to D.

**Figure 4:**
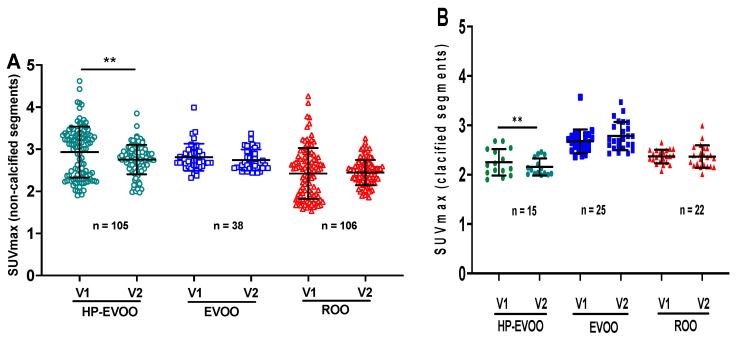
Comparison of the SUVmax values for ^18^F-FDG at baseline and after 6-month supplementation with three different olive oils for (**A**) the non-calcified and (**B**) calcified segments of the aorta. ** *p* < 0.02.

Figure 4 shows the quantitative data on ^18^F-FDG uptake in non-calcified segments of the aorta at baseline and after the 6-month supplementation. Interestingly, HP-EVOO induced a significant decrease in ^18^F-FDG uptake in the aorta (2.93 ± 0.23 to 2.75 ± 0.38, *p* < 0.004). However, no significant changes were observed between baseline and the 6-month supplementation with EVOO (2.81 ± 0.40 and 2.74 ± 0.31, respectively) or ROO (2.41 ± 0.66 and 2.44 ± 0.28, respectively) (Figure 4A). ^18^F-FDG uptake was also quantified in the calcified segments of the aorta. HP-EVOO intake induced a significant reduction in ^18^F-FDG uptake in calcified segments of the aorta compared to the non-calcified segments (2.25 ± 0.29 and 2.15 ± 0.19, respectively, *p* < 0.02), whereas no significant changes were observed with EVOO or ROO (Figure 4B).

Panel (A) was SUVmax for non-calcified slices and panel (B) was SUVmax for calcified slices. The values were determined at baseline and after 6 months of olive oil supplementation for each participant. The data were obtained from transaxial image slices. The letter n represents the number of artery slices evaluated for ^18^F-FDG uptake. *p* < 0.05 was considered significant. HP-EVOO: high polyphenol extra virgin olive oil; EVOO: extra virgin olive oil; ROO: refined olive oil.

**Figure 5:**
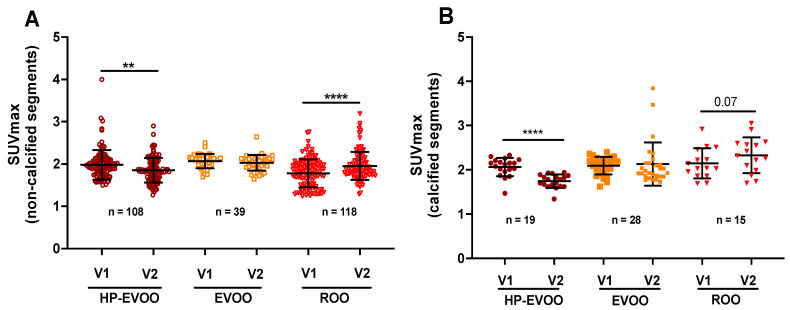
Comparison of the SUVmax values for ^18^F-NaF uptake at baseline and after 6-month supplementation with three different olive oils for (**A**) the non-calcified and (**B**) calcified segments of the aorta. ** *p* < 0.002 and **** *p* < 0.0001.

Figure 5 shows the effect of olive oil supplementation on microcalcification as evaluated by ^18^F-NaF uptake. Our results showed that the 6-month supplementation with HP-EVOO induces a significant reduction in ^18^F-NaF uptake (SUVmax = 1.98 ± 0.33 at baseline compared to 1.85 ± 0.28, after the 6-month supplementation, *p* < 0.004) (Figure 5A). No significant changes were observed for EVOO supplementation (2.06 ± 0.21 vs. 2.02 ± 0.20). Conversely, ROO supplementation for 6 months resulted in a significant increase in ^18^F-NaF uptake (SUVmax = 1.78 ± 0.34 at baseline and 1.95 ± 0.34 after the 6-month supplementation with ROO, *p* < 0.0001) (Figure 5A). Figure 5B presents data related to microcalcification in the calcified segments of the aorta. As observed for the non-calcified segments, the 6-month supplementation with HP-EVOO induced a significant reduction in ^18^F-NaF uptake in the calcified segments of the aortas (SUVmax = 2.06 ± 0.25 compared to 1.74 ± 0.15, *p* < 0.0001). No significant effect was observed for EVOO, whereas an increasing trend for ^18^F-FDG uptake was observed after the 6-month supplementation period with ROO (Figure 5B).

Panel (A) was SUVmax for non-calcified slices and panel (B) was SUVmax for calcified slices. The values were determined at baseline and after 6 months of olive oil supplementation for each participant. The data were obtained from transaxial image slices. n represents the number of artery slices evaluated for ^18^F-NaF uptake. *p* < 0.05 was considered significant. HP-EVOO: high polyphenol extra virgin olive oil; EVOO: extra virgin olive oil; ROO: refined olive oil.

Table 3 summarizes the SUVmax values ^18^F-FDG and ^18^F-NaF and for both non-calcified and calcified segments of the aorta, at baseline and after the 6-month intervention with the three different olive oils used.

## 4. Discussion

Atherosclerosis, a chronic condition characterized by the buildup of cholesterol plaques on arterial walls leading to arterial stiffening, is considered a disease of the elderly [30]. Inflammation is an important contributor to the development of atherosclerosis and the occurrence of clinical events. Anti-inflammatory therapy is associated with a significant reduction in the incidence of severe cardiovascular events in patients at high cardiovascular risk [31]. However, few studies have focused on inflammation associated with age (inflammaging) and its impact on atherosclerosis risk. The chronic low-grade inflammation that characterizes the process of aging, combined with oxidative stress, contributes to vascular damage, and increases the development of the atherosclerotic process. There is thus a pressing need for effective strategies to detect atherosclerotic disease in its early stages and to quantify the disease burden in the elderly.

The ^18^F-FDG radiotracer for metabolic activity is increasingly being used as a probe to assess atherosclerosis progression and atherosclerotic plaque stability. Several studies have shown that there is a significant correlation between ^18^F-FDG uptake and carotid lesions, suggesting that high ^18^F-FDG uptake could be used as a marker of the early stages of the atherosclerotic process. The results of the present study, which was conducted with healthy elderly subjects with no signs of CVD and, except for aging, with no other CVD risk factor, showed that the aorta, while apparently healthy (non-calcified), takes up a large amount of ^18^F-FDG. Intriguingly, these non-calcified segments displayed significantly higher ^18^F-FDG uptake than the calcified segments of the aorta. This highlights the ability of ^18^F-FDG to localize arterial inflammation at the early stages of atherosclerotic lesion formation before calcification. Indeed, although calcification is considered an advanced stage of the atherosclerotic process, previous studies have not found a correlation between ^18^F-FDG uptake levels and CT calcium scores [32]. The ^18^F-FDG radiotracer is a glucose analogue that preferentially accumulates in M1 phenotype proinflammatory macrophages that initiate and sustain inflammation. Of note, the mean SUVmax of the imaged participants in the present study were 2.70 ± 0.62 to 2.54 ± 0.44 in the non-calcified and calcified segments of the aorta, respectively. These values are slightly higher than those obtained with younger cardiovascular patients presenting at least one risk factor for CVD (mean SUVmax = 2.14 ± 0.37) [33]. This indicates that the high uptake of ^18^F-FDG may be a consequence of damage induced at the vascular level by the dominance of a low-grade inflammatory state due to the relatively advanced age of our participants. Indeed, inflammaging has been reported to induce significant changes in vascular structure and imbalances in immune homeostasis [34]. Nevertheless, our SUVmax values were still significantly lower than those of patients suffering from elevated vascular inflammation such as vasculitis (mean SUVmax = 3.12 to 4.05, respectively, for the thoracic and abdominal aortas) [35].

The inflammatory process of atherosclerotic lesion formation triggers the process of vascular calcification. M1 macrophages secrete inflammatory cytokines (interleukin-6, interleukin-1 β, and tumor necrosis factor (TNF)-α) that induce the osteoblastic transformation of vascular smooth muscle cells, promoting the formation of hydroxyapatite structures in atherosclerotic plaques [36]. Although dense calcified plaques have traditionally been qualified as more stable [37], microcalcification (0.5–50 μM) is an early step in the continuum of the process leading to the calcification of atherosclerotic plaques [38] and is a marker for plaque fragility. ^18^F-NaF has been shown to be valuable for identifying areas displaying microcalcification, even in CT-negative calcification regions of human atherosclerotic lesions [39]. Our results revealed the presence of microcalcification in both the non-calcified and calcified segments of the aorta. This microcalcification was significantly higher in the calcified segments of the aorta than in the non-calcified segments (SUVmax = 2.09 ± 0.24 and 1.90 ± 0.4, respectively, *p* < 0.0001). These results are in accordance with a previous study [27].

The effective management of risk factors and the adoption of a healthy lifestyle are pivotal in mitigating or preventing atherosclerotic lesion formation and atherosclerosis progression. Olive oil consumption is strongly associated with a reduced risk for CVD [40,41]. Although the exact mechanisms of this protection have not been clearly elucidated, several studies have suggested that polyphenols, Tyr and HTyr in particular, play a significant protective role [42,43] in reducing oxidative stress and preventing LDL peroxidation, which is a key step in the initiation of atherosclerotic plaque formation [44]. Other studies have also shown that Tyr and HTyr exert a vasculo-protective effect by downregulating vascular cell adhesion molecules [45]. In the present study, we studied the effect of three olive oils with different levels of Tyr and HTyr on arterial inflammation and subsequent stages, notably microcalcification. HP-EVOO, which has a much higher Tyr and HTyr content than the other two olive oils, produced a substantial reduction in ^18^F-FDG uptake in both non-calcified and calcified arteries. However, the measurement of systemic biomarkers of inflammation did not reveal any significant changes or improvements over the 6-month supplementation period for any of the three olive oils used. This highlighted the high sensitivity of ^18^F-FDG PET imaging for detecting arterial inflammation, even at systemic inflammation levels too low to be detected by the usual inflammatory biomarkers.

HP-EVOO also significantly reduced microcalcification in both the non-calcified and calcified segments of the aorta. In contrast, 6 months of ROO supplementation was accompanied by an increase in arterial microcalcification, as shown by the increase in ^18^F-NaF uptake in the non-calcified segments of the aorta. Although, unlike ROO, EVOO did not appear to induce a significant change in arterial microcalcification, it stabilized microcalcification levels throughout the 6-month supplementation period.

The seven-country study showed that there was low cardiovascular mortality in the Mediterranean region compared to other countries after a 50-year follow-up of approximately 12,000 participants [46]. An epidemiological study attributed the significant anti-atherosclerotic effect of this diet to its high olive oil content [47]. Much attention has been paid to phenolic compounds as a whole [48]. Tyr and HTyr are the two major phenolic compounds that naturally occur in olives and olive oil. These simple phenolic compounds have been shown to possess significant antioxidant activity and to prevent LDL lipid peroxidation, the first step in the atherosclerotic process [49]. Other studies have shown that Tyr and HTyr possess anti-inflammatory activity [50] and reduce inflammaging-associated damage [51]. Our results are the first to show that Tyr and HTyr reduce arterial inflammation and microcalcification formation in a healthy elderly population. However, this effect becomes significant only with relatively high levels of Tyr and HTyr intake. The European Food Safety Authority (EFSA) and the European Commission have set the amount of olive oil to be consumed daily to produce a beneficial effect on health at 20 mL, on the condition that it provides a minimum 5 mg daily intake of Tyr and HTyr [52]. However, the Tyr and HTyr content is quite low in most olive oils (a maximum of 2 to 14 mg/kg of Tyr and HTyr in regular extra virgin olive oil) [53,54] for an average intake of 0.28 mg/day of these phenolic compounds. The HP-EVOO used in the present study, due to its high content in Tyr and HTyr, makes possible an average intake of 7.12 mg/day of these two phenolic compounds. Although this intake level is above the recommended level needed to produce a beneficial effect, it is associated with a significant reduction in arterial inflammation and microcalcification. Nevertheless, regular HP-EVOO consumption, even at amounts below the recommended level, still stabilized inflammation and microcalcification levels over the 6-month study period. This implies that the quantity of Tyr and HTyr in HP-EVOO protects against the development of CVD and the incidence of cardiovascular events by, in part, stabilizing arterial inflammation. However, greater Tyr/HTyr intake should be recommended in patients at high cardiovascular risk, particularly those with a high-inflammatory status (diabetes, hypercholesterolemia, etc.).

Notably, several studies have shown that therapeutic interventions such as statins can curtail ^18^F-FDG uptake at lesion sites and thus decrease arterial inflammation [17,55]. However, all these interventions are used for secondary prevention in patients at high risk for CVD or at risk of a cardiovascular event [17,56]. To the best of our knowledge, our study is the first to investigate the effect of a nutritional intervention on arterial inflammation and vascular macrocalcification in healthy elderly subjects. HP-EVOO caused a significant reduction in both arterial inflammation and microcalcification and, as such, our results may open a new therapeutic avenue by targeting personalized dietary supplementation with Tyr and HTyr for high-risk CVD patients.

## 5. Limitations and Directions for Future Studies

The present study had several limitations due to the small number of participants, which affected its statistical power. Nonetheless, our findings shed light on how the composition of olive oil impacts arterial inflammation and microcalcification. Importantly, the present study is one of the first to demonstrate the effect of high Tyr and HTyr intake on vascular health using state-of-the-art imaging technology. Further research with larger and more diverse participant groups with respect to CVD risk factors is needed to elucidate the mechanisms underlying these compelling findings.

## 6. Conclusions

Atherosclerosis, which is characterized by arterial plaque buildup, is a significant global health concern. The present study showed that ^18^F-FDG/^18^F-NaF- PET/CT imaging displays remarkable sensitivity for detecting arterial inflammation and plaque microcalcification in heathy elderly patients and for assessing the effect of nutritional interventions on these two markers of atherosclerotic lesions and plaque stability. Our results also showed that a high intake of olive oil phenolic compounds (Tyr and HTyr) significantly reduces arterial inflammation in healthy and calcified arteries and decreases the microcalcification process in atherosclerotic lesions and plaques. Our findings showed that advanced imaging techniques are effective in identifying vascular lesions before calcified plaques develop and offer a promising avenue for early detection and prevention in cardiovascular health. The present study also shed light on the beneficial effect of phenolic compounds in terms of protecting against CVD.

## Figures and Tables

**Table 1:** Anthropometric and biochemical characteristics of participants at baseline (V1) and after 6-month intervention (V2) with three different olive oils.

Parameters	HP-EVOO		EVOO		ROO	
	V1	V2	V1	V2	V1	V2
Numbers of participants	(2M; 1F)		(2M; 1F)		(2M; 3F)	
Age	72 ± 5.48		79 ± 0.58		78 ± 5.66	
Weight (kg)	94.36 ± 34.73	103.37 ± 38.55	65.80 ± 18.54	66.40 ± 16.44	80.36 ± 13.98	83.6 ± 10.16
BMI	32.28 ± 10	34.78 ± 11.42	24.86 ± 3.10	25.17 ± 2.32	28.55 ± 5.10	29.77 ± 4.37
PA systolic	139.67 ± 16.17	140.67 ± 15.04	125.50 ± 19.09	123.33 ± 17.21	149.80 ± 24.01	145.40 ± 32.47
PA diastolic	83.33 ± 3.21	83.67 ± 3.79	74.00 ± 11.31	72.67 ± 14.50	82.12 ± 7.18	80.60 ± 12.97
Total cholesterol (mmol/L)	5.06 ± 0.54	4.9 ± 0.76	4.64 ± 0.24	5.15 ± 0.41	4.67 ± 0.74	4.74 ± 1.35
C-HDL (mmol/L)	1.64 ± 0.43	1.59 ± 0.37	1.46 ± 0.59	1.57 ± 0.72	1.67 ± 0.43	1.69 ± 0.51
C-LDL (mmol/L)	2.9 ± 0.37	2.83 ± 0.53	2.68 ± 0.53	2.97 ± 0.35	2.59 ± 0.59	2.65 ± 0.97
CRP (mg/L)	2.20 ± 2.34	2.95 ± 4.10	1.80 ± 0.66	2.50 ± 1.47	1.24 ± 0.61	1.31 ± 0.42
Lp(a) (nmol/L)	21.03 ± 24.48	41.57 ± 7.72	82.60 ± 130.51	135.25 ± 180.10	90.48 ± 50.64	88.66 ± 43.07
Hemoglobin A1c (%)	5.50 ± 0.35	5.53 ± 0.23	5.93 ± 0.06	6.10 ± 0.10	5.50 ± 0.27	5.50 ± 0.22
Troponin T (ng/L)	7.33 ± 3.06	10.12 ± 4.58	10.67 ± 7.23	12.33 ± 8.39	12 ± 1.22	13.60 ± 0.55
TSH (mUI/L)	2.06 ± 0.03	2.12 ± 0.23	1.89 ±1.07	2.72 ± 0.71	3.81 ± 4.59	2.18 ± 0.93
Fibrinogen (g/L)	2.94 ± 0.99	2.95 ± 0.68	3.30 ± 0.66	3.03 ± 0.28	2.75 ± 0.13	3.27 ± 0.59

HP-EVOO: High-polyphenol extra virgin olive oil, EVOO: extra virgin olive oil, ROO: refined, olive oil, BMI: body mass index, CPR: C-Protein reactive, C-HDL: high-density lipoprotein cholesterol, C-LDL: low-density lipoprotein cholesterol, PA: arterial pressure, THS: thyroid stimulating hormone.

**Table 2:** The levels of phenolic compounds in the three olive oils used for supplementation.

	ROO (mg/kg)	EVOO (mg/kg)	HP-EVOO (mg/kg)
Total phenolic compounds	2.7	255	1 249
Tyrosol	n.d.	6.3	123.1
Hydroxytyrsol	n.d.	7.8	233.6

ROO: refined olive oil. EVOO: extra virgin olive oil, HP-EVOO: high-polyphenol extra virgin olive oil, n.d.: non-detectable.

**Table 3:** SUVmax values for ^18^F-FDG and ^18^F-NaF for both non-calcified and calcified segments of the aorta, at baseline and after the 6-month intervention with different olive oils.

Parameters	HP-EVOO		*p*	EVOO		*p*	ROO		*p*
	V1	V2		V1	V2		V1	V2	
Non-calcified segments of the aorta									
SUVmax ^18^F-FDG	2.93 ± 0.23	2.75 ± 0.38	0.004	2.81 ± 0.40	2.74 ± 0.31	ns	2.41 ± 0.66	2.44 ± 0.28	ns
SUVmax ^18^F-NAF	1.98 ± 0.33	1.85 ± 0.28	0.004	2.06 ± 0.21	2.02 ± 0.20	ns	1.78 ± 0.34	1.95 ± 0.34	<0.0001
Calcified segments of the aorta									
SUVmax ^18^F-FDG	2.25 ± 0.29	2.15 ± 0.19	0.004	2.67 ± 0.27	2.78 ± 0.30	ns	2.36 ± 0.10	2.36 ± 0.23	ns
SUVmax ^18^F-NAF	2.06 ± 0.25	1.74 ± 0.15	0.0001	2.09 ± 0.17	2.12 ± 0.49	ns	2.13 ± 0.35	2.32 ± 0.40	<0.0001

HP-EVOO: High-polyphenol extra virgin olive oil, EVOO: extra virgin olive oil, ROO: refined olive oil. V1: visit 1 (baseline); V2: visit 2 (6-month supplementation with olive oil). ns: nonsignificant

## Data Availability

Data are contained within the article.
